# Combined Evaluation of MAP1LC3B and SQSTM1 for Biological and Clinical Significance in Ductal Carcinoma of Breast Cancer

**DOI:** 10.3390/biomedicines9111514

**Published:** 2021-10-21

**Authors:** Pei-Feng Liu, Chih-Wen Shu, Hsiu-Chen Yang, Cheng-Hsin Lee, Huei-Han Liou, Luo-Ping Ger, Yen-Dun Tony Tzeng, Wen-Ching Wang

**Affiliations:** 1 Department of Biomedical Science and Environmental Biology, College of Life Science, Kaohsiung Medical University, Kaohsiung 80708, Taiwan; pfliu@kmu.edu.tw (P.-F.L.); R980084@kmu.edu.tw (C.-H.L.); 2 Department of Medical Research, Kaohsiung Medical University Hospital, Kaohsiung 80708, Taiwan; 3 Center for Cancer Research, Kaohsiung Medical University, Kaohsiung 80708, Taiwan; 4 Institute of Biomedical Sciences, National Sun Yat-sen University, Kaohsiung 80424, Taiwan; 5 Institute of BioPharmaceutical Sciences, National Sun Yat-sen University, Kaohsiung 80424, Taiwan; cwshu@g-mail.nsysu.edu.tw; 6 Department of Medical Education and Research, Kaohsiung Veterans General Hospital, Kaohsiung 81362, Taiwan; mini16610@gmail.com (H.-C.Y.); hhliou@vghks.gov.tw (H.-H.L.); lpger0329@gmail.com (L.-P.G.); 7 Department of Surgery, Kaohsiung Veterans General Hospital, Kaohsiung 81362, Taiwan; 8 Department of General Surgery, Chi Mei Medical Center, Tainan 71004, Taiwan

**Keywords:** invasive ductal carcinoma, MAP1LC3B, SQSTM1, autophagy inhibition, chemoresistance, prognosis

## Abstract

Breast cancer is the leading cause of cancer death in women worldwide. The microtubule-associated protein light chain 3B (MAP1LC3B) and adaptor sequestosome 1 (SQSTM1) are two major markers for autophagy. Increased protein levels of MAP1LC3B and SQSTM1 are considered to be causes of autophagy inhibition or activation in various types of cancers. However, the roles of MAP1LC3B and SQSTM1 in breast cancer are still not clear. Using a tissue microarray from 274 breast invasive ductal carcinoma (IDC) patients, we found that tumor tissues showed higher protein levels of MAP1LC3B and cytoplasmic SQSTM1 in comparison to those in adjacent normal tissues. Moreover, high levels of MAP1LC3B were associated with better survival, including disease-specific survival and disease-free survival (DFS) in IDC patients. Furthermore, high co-expression of MAP1LC3B and SQSTM1 was significantly associated with better DFS in IDC patients. Astonishingly, the autophagy inhibitor accumulated the protein levels of MAP1LC3B/SQSTM1 and enhanced the cytotoxic effects of cisplatin and paclitaxel in MCF7 and BT474 breast cancer cell lines, implying that autophagy inhibition might result in poor prognosis and chemosensitivity in IDC. Taken together, high co-expression of MAP1LC3B and SQSTM1 might serve as a potential diagnostic and prognostic biomarker for IDC patients.

## 1. Introduction

Breast cancer is the most prevalent type of malignancy among women and a major leading cause of cancer death worldwide. Breast invasive ductal carcinoma (IDC), a common type of breast cancer, comprises nearly 70–80% of all breast cancer [1]. Traditional classification systems in biological characteristics of molecular subtype specific proteins may have limitations for breast cancer patient-tailored treatment [2], suggesting the urgent need for effective diagnostic and prognostic biomarkers in breast cancer patients.

Autophagy is crucial for physiological homeostasis, but its dysfunction causes many diseases including cancer, neurodegenerative diseases and infection. Moreover, autophagy serves as a “double-edged sword” with opposite functions in tumor initiation and malignancy [3]. Most studies have indicated that autophagy activation is implicated in tumor progression [3,4]. Thus, autophagy could be a therapeutic target in cancer [5]. In contrast, autophagy defects could cause sensitivity to metabolic stress, DNA damage accumulation, genomic instability and accelerated tumorigenesis in some cancers [6].

The microtubule-associated light chain 3B (MAP1LC3B) acts as an autophagy receptor for autophagosome elongation in mammalian cells [7]. High expression of MAP1LC3B is associated with poor survival [3] but also has better outcomes in patients with different types of cancer [8]. On the other hand, SQSTM1 (also known as sequestosome 1; hereafter referred to as p62) with MAP1LC3B-interacting and ubiquitin-associated domains serves as an autophagic adaptor protein for recruiting ubiquitinated proteins into the autophgosome [9]. Autophagy-defective tumor cells preferentially accumulate SQSTM1, and higher expressions of SQSTM1 have been detected in several cancers including prostate [10], breast [11], kidney [12], liver [13], lung [14], breast [15], oral [16], colon [11], ovarian [17], head and neck [11] and gastric cancers [11]. Elevated spontaneous tumor formation and tumor progression have been found to be accompanied by SQSTM1 accumulation [18]. Moreover, the degree of SQSTM1 expression is significantly correlated with aggressive clinicopathologic features such as distant metastasis [19]. Higher expression of SQSTM1 is related to worse prognosis [8] and higher recurrence of several types of tumors [20]. These findings imply that SQSTM1 plays different roles in various types of cancer and could be a potential target for cancer therapy [16]. However, little is known about the role MAP1LC3B and SQSTM1 co-expression plays in breast cancer.

In this study, we compare protein levels of both MAP1LC3B and SQSTM1 between tumor tissues and corresponding tumor adjacent normal (CTAN) tissues in IDC patients. Moreover, we investigate the prognostic role of MAP1LC3B/SQSTM1 co-expression in IDC patients. In addition, the accumulated MAP1LC3B and SQSTM1 proteins in breast cancer cell lines treated with autophagy inhibitors and their role in chemoresistance of breast cancer cell lines are studied. Our study is the first to suggest the biological role and clinical significance of the MAP1LC3B/SQSTM1 co-expression in IDC patients.

## 2. Materials and Methods

### 2.1. Tissue Specimens

Margin-free (margin-size ≥ 0.2 cm) paraffin-embedded materials of CTAN and tumor tissues were obtained from 274 IDC patients. Clinicopathological data including age, pathological stage, TNM classification and cell differentiation were collected from patient records. TNM classification was determined at the time of initial tumor resection in accordance with the guidelines of the 2002 American Joint Committee. Written informed consent was not required as all data and specimens were previously collected and analyzed anonymously. This study was approved by the Institutional Review Board of the Kaohsiung Veterans General Hospital (VGHKS12-CT2-07) and was conducted following the Helsinki declaration.

### 2.2. Tissue Microarray (TMA) Construction

All paraffin-embedded tissues were used for TMA block construction. A TMA block consists of 48 trios; each trio contains one core with CTAN tissue and two cores with tumor tissue from each IDC patient. Each TMA block was cut into 4 μm-thick paraffin sections for further immunohistochemical staining [1].

### 2.3. Immunohistochemistry (IHC)

The cut paraffin sections were dewaxed in xylene, rehydrated through a series of graded alcohols (100%, 95%, 75%, 50% and 30%) and then washed for 5 min with phosphate-buffered saline (PBS). The Novolink max polymer detection system (Leica, Newcastle Upon Tyne, UK) was used for immunostaining. Antigen retrieval was performed by immersion in Tris-EDTA (10 mM, PH 9.0) for MAP1LC3B and sodium citrate for SQSTM1 (10 mM, PH 6.0) for 10 min at 125 °C, respectively. Endogenous peroxidase activity was blocked using 3% hydrogen peroxide in methanol at room temperature for 30 min. After blocking, the slides were incubated in a wet chamber with cytoplasmic SQSTM1 rabbit polyclonal antibody (dilution 1:1000; Enzo Life Sciences, Farmingdale, NY, USA) and MAP1LC3B (dilution 1:100; NanoTools, San Diego, CA, USA) at 4 °C overnight. Color development was performed using 0.03% diaminobenzidine, and hematoxylin was used for counterstaining [3].

### 2.4. IHC Scoring

A semi-quantitative approach based on staining intensity and percentage was used to score the degree of immune reactivity for IHC. The scores of staining intensity and percentage of the positive cells were added to obtain final scores ranging from 0 to 7. IHC scoring procedures were described in greater detail in our previous study [21].

### 2.5. Cell Culture

Two breast cancer cell lines, MCF-7 [(human epidermal growth factor receptor 2 (HER2)-/estrogen receptor(ER)+/progesterone receptor(PR)+) and BT474 (HER2^+^/ER^+^/PR^+^), were cultured in Corning tissue culture-treated plastic (Corning, Inc., Corning, NY, USA) containing DMEM/F12 (Gibco, Invitrogen Corporation, Carlsbad, CA, USA) with 10% FBS, 100 μg/mL streptomycin, 100 U/mL penicillin and 1% L-glutamine at 37 °C with 5% CO_2_: 95% air.

### 2.6. Western Blotting

Cell lines pre-treated with bafilomycin A1 (BafA1, 100 nM, Selleckchem, Houston, TX, USA), concanamycin A (ConA, 10 nM, Merck & Co., Inc., Kenilworth, NJ, USA) and chloroquine (CQ, 20 μM, Sigma-Aldrich Corporation, St. Louis, MO, USA) were lysed with a RIPA buffer containing 0.1% SDS, 150 mM NaCl, 50 mM Tris-Cl pH7.5, 1% NP40, 0.25% sodium deoxycholate and a protease inhibitor cocktail. The different protein molecules in the cell lysates were separated by SDS–PAGE and then transferred to nitrocellulose membranes. The membrane was incubated with the primary antibodies at 4 °C overnight after blocking with 5% skim milk and then probed with HRP-labeled secondary antibody. Afterwards, the membrane was detected with ECL reagent, and the protein level was analyzed using the BioSpectrum^®^ Imaging System (UVP, Inc., Upland, CA, USA).

### 2.7. Tumor Sphere Formation and Sphere Cell Viability

The cell lines (5 × 10^3^/mL) pre-treated with autophagy inhibitors (20 μM CQ) for 5 h were seeded into a 96-well clear round-bottom ultra-low attachment Microplate (Corning Costar, Cambridge, MA, USA) to form cell spheroids that were then treated with or without 50 μM cisplatin (CIS) (Sigma-Aldrich Corporation, St. Louis, MO, USA) or 0.05 μM PTX (Selleckchem, Houston, TX, USA) for 24 h. The CellTiterGlo 3D system (Promega, Madison, WI, USA) was then used to measure sphere cell viability [22].

### 2.8. Live/Dead Cell Viability Assay

Cell lines (5 × 10^3^ mL) were first pre-treated with autophagy inhibitors (20 μM CQ) for 5 h then cultured in Nano Culture Plates (NCPs; MBL Corporation, Ottawa, llinois, USA) for formation of cell spheroids. Afterwards, spheroid cells were treated with or without 50 μM CIS or 0.05 μM PTX for 24 h. Subsequently, the live/dead cell viability assay (LIVE/DEAD^®^ Viability/Cytotoxicity Kit, Thermo Fisher Scientific, Waltham, MA, USA) was used for analyzing the cell viability. The live (green)/dead (red) spheres were observed by fluorescence microscopy, and the cell viability was quantitated by a Fluoroskan Ascent FL reader [23].

### 2.9. Statistical Analysis

The different protein levels between CTAN and the tumor tissues were analyzed using the Wilcoxon signed-rank test. The correlation between protein levels and clinicopathologic parameters were evaluated using a Student’s t test, Mann–Whitney U test, Kruskal–Wallis one-way ANOVA test and one-way ANOVA test. Disease-specific survival (DSS) was calculated as the date from the initial resection of the primary tumor to the date of cancer-specific death or last follow-up. Disease-free survival (DFS) was calculated as the date of the primary tumor resection to the date of recurrence or last follow-up. For survival analysis, the high and low levels of SQSTM1 and MAP1LC3B were dichotomized using cutoffs of 50%. A two-tailed *p*-value ≤ 0.05 was considered statistically significant.

## 3. Results

### 3.1. Comparison of MAP1LC3B and SQSTM1 Expressions between CTAN and Tumor Tissues in IDC Patients

**Figure 1:**
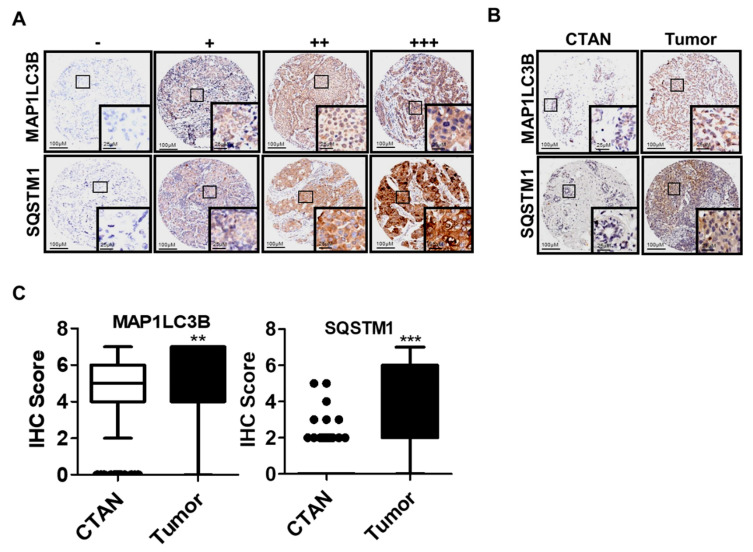
Comparison of MAP1LC3B and SQSTM1 expressions in IDC patients. (**A**) Protein expression of MAP1LC3B and cytoplasmic SQSTM1 were measured by IHC and staining intensity was categorized as − = no expression; + = weak expression; ++ = moderate expression; +++ = strong expression (10× and 40× magnification). (**B**) Representative immunoreactivity intensity of MAP1LC3B and SQSTM1 between CTAN and tumor tissues of one paired IDC patients (10× and 40× magnification). (**C**) Range of IHC scores of MAP1LC3B and SQSTM1 between CTAN tissues and tumor tissues, respectively, from 91 to 81 paired IDC patients (N: CTAN tissues; T: tumor tissues). ** *p* < 0.01; *** *p* < 0.001.

We analyzed the protein levels of MAP1LC3B and SQSTM1 in tissues of IDC patients by IHC staining. The scores of staining intensity for SQSTM1 and MAP1LC3B were measured using a numerical scale (−, no expression; +, weak expression; ++, moderate expression; and +++, strong expression, Figure 1A). We found that protein levels of MAP1LC3B (4.41 ± 2.18 vs. 5.14 ± 1.84, *p* = 0.001, Table 1) and SQSTM1 (0.51 ± 1.16 vs. 3.84 ± 2.24, *p* <0.001, Table 1) were higher in the tumor tissue than in the CTAN tissues of IDC patients (Figure 1B,C). These results indicate that the levels of MAP1LC3B and SQSTM1 in tumor tissues were different from those in CTAN tissues of IDC patients.

### 3.2. Correlation of MAP1LC3B and SQSTM1 Expressions with Clinicopathological Outcomes and Prognosis in IDC Patients

To further investigate the clinical role of MAP1LC3B and SQSTM1 in IDC patients, the correlation of MAP1LC3B and SQSTM1 expression with clinicopathological outcomes in IDC breast patients was analyzed. We found that only high protein levels of MAP1LC3B were correlated with poor cell differentiation (*p* = 0.045, Table 2) in IDC patients. Moreover, we analyzed the correlation of MAP1LC3B and SQSTM1 expressions with DSS and DFS in IDC patients. After adjusting for AJCC pathological stage (II and III vs. I), grading (III vs. I and II), adjuvant treatment (incomplete or inappropriate vs. non-treatment or complete) and molecular subtypes (luminal B, HER2 over-expression and basal-like vs. luminal A) by multivariate Cox’s regression, only high protein levels of MAP1LC3B were correlated with better DSS (AHR: 0.64, 95% CI: 0.44–0.94, *p* = 0.022, Table 3) and DFS (AHR: 0.54, 95% CI: 0.36–0.81, *p* = 0.003, Table 3) in IDC patients. Remarkably, IDC with high co-expression of MAP1LC3B and SQSTM1 had a lower hazard factor and better DFS (AHR: 0.51, 95% CI: 0.32–0.81, *p* = 0.005, Table 4). These results indicate that high co-expression of MAP1LC3B and SQSTM1 was correlated with better DFS in IDC patients.

### 3.3. Protein Levels of MAP1LC3B and SQSTM1 in Breast Cancer Cell Lines

**Figure 2:**
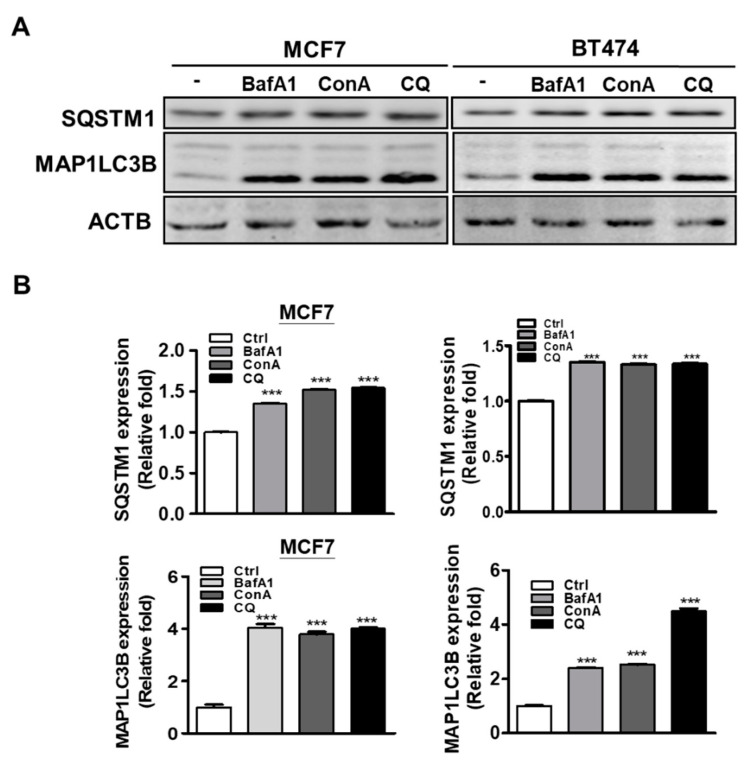
Effects of autophagy inhibitors on expressions of MAP1LC3B and SQSTM1 in breast cancer cell lines. (**A**) Evaluation of MAP1LC3B and SQSTM1 expressions in MCF7 and BT474 cell lines treated with autophagy inhibitors (BafA1 (100 nM), ConA (10 nM) and CQ (20 μM)) for 5 h by western blotting. (**B**) Levels of MAP1LC3B and SQSTM1 protein in MCF7 and BT474 cell lines treated with autophagy inhibitors. Results represent the mean of three independent experiments. *** *p* < 0.001.

To confirm that MAP1LC3B and SQSTM1 expressions were up-regulated due to autophagy inhibition, breast cancer cell lines (MCF7 and BT474) were treated with or without autophagy inhibitors [BafA1(100 nM), ConA (10 nM) and CQ (20μM)] for 5 h before harvesting. Accumulated protein levels of MAP1LC3B and SQSTM1 in treated breast cancer cell lines were observed (Figure 2A) and quantified (Figure 2B). These results indicate that the accumulation of MAP1LC3B and SQSTM1 might be caused by autophagy inhibition in breast cancer cell lines.

### 3.4. Effect of Autophagy Inhibition on Chemoresistance in Breast Cancer Cell Line

**Figure 3:**
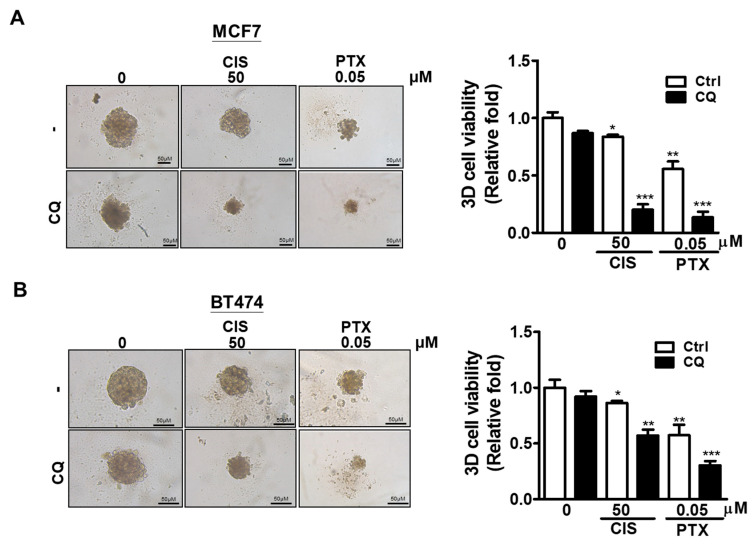
Effects of autophagy inhibition on tumorsphere formation and sphere cell viability in breast cancer cell lines treating cancer drugs. (**A**) MCF7 and (**B**) BT474 cell lines pre-treated with autophagy inhibitor CQ (20 μM) for 5 h were cultured in a round bottom ultra-low plate for tumorsphere formation. The tumorspheres were then treated with or without cisplatin (CIS, 50 μM) or paclitaxel (PTX, 0.05 μM) for 24 h. The sphere morphology and cell viability were detected under light microscopy (100× magnification) and using the CellTiterGlo 3D system, respectively Results represent the mean of three independent experiments. * *p* < 0.05; ** *p* < 0.01; *** *p* < 0.001.

**Figure 4:**
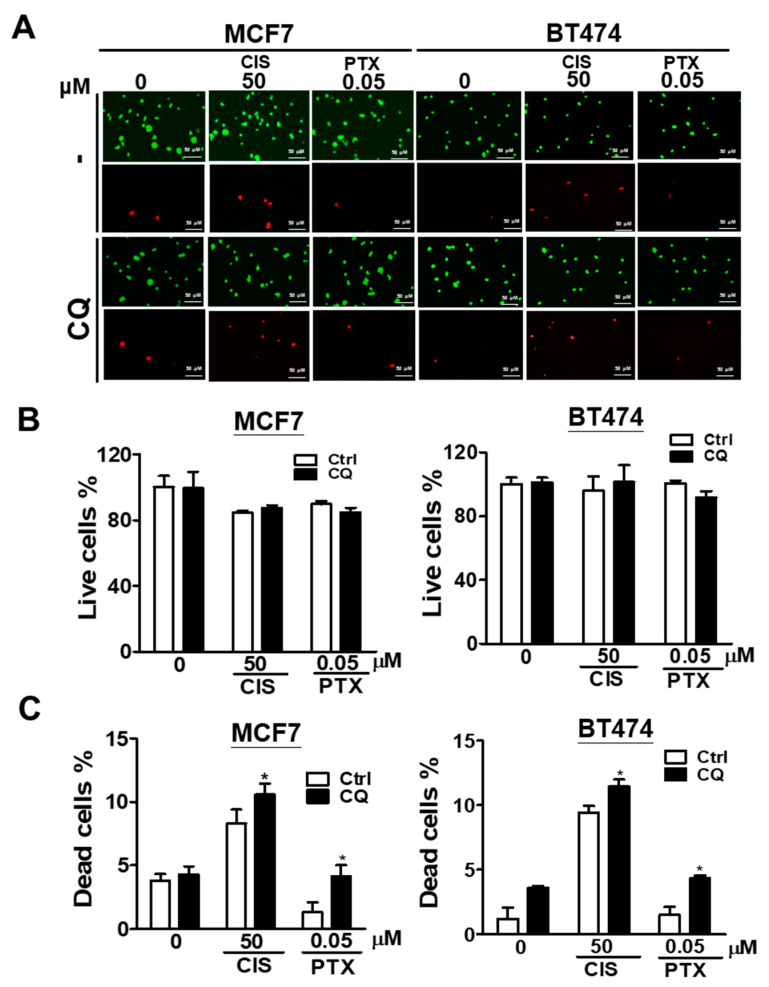
Effects of autophagy inhibition on live/dead cell viability in breast cancer cell lines treating anti-cancer drugs. Breast cancer cell lines pre-treated with autophagy inhibitor CQ (20 μM) for 5 h were cultured on NanoCulture plates for tumorsphere formation. The tumorsphere was then treated with cisplatin (CIS, 50 μM) or paclitaxel (PTX, 0.05 μM) for 24 h. (**A**) The viable (green) and dead (red) cells were assayed using a LIVE/DEAD staining kit and observed by the fluorescence microscopy (40× magnification). The (**B**) viable and (**C**) dead cells were quantified by a Fluoroskan Ascent FL reader. Results represent the mean of three independent experiments * *p* < 0.05.

In the study, higher protein levels of MAP1LC3B and SQSTM1 were found in tumor tissues, and the high co-expression of MAP1LC3B and SQSTM1 was associated with tumorigenesis and better DFS. Moreover, we found that MAP1LC3B and SQSTM1 might be accumulated due to autophagy inhibition in breast cancer cell lines. To determine the effects of autophagy inhibition on chemoresistance in IDC, a tumorsphere culture model and Live/Dead assay for drug resistance were used. Interestingly, MCF7 (Figure 3A) and BT474 (Figure 3B) cell lines pre-treated with autophagy inhibitor CQ showed decreased tumorsphere formation and enhanced chemosensitivity to CIS and PTX in a dose-independent manner (Figure S1). Moreover, the Live/Dead staining assay (Figure 4A) indicated that autophagy inhibitor CQ slightly reduced live MCF7 and BT474 cells (Figure 4B) but significantly increased dead MCF7 and BT474 cells (Figure 4C) in the presence of CIS and PTX. Taken together, these results indicate that accumulated MAP1LC3B and SQSTM1 caused by autophagy inhibition might be involved in the chemoresistance of breast cancer cell lines.

## 4. Discussion

MAP1LC3B and SQSTM1 are essential to the autophagy machinery. The accumulation of MAP1LC3B and SQSTM1 plays a different role in clinicopathological outcomes and prognosis in various types of cancer [3]. However, the biological role and clinical significance of MAP1LC3B and SQSTM1 in breast cancer remains unclear. We found that protein levels of MAP1LC3B and SQSTM1 were higher in tumor tissues than in CTAN tissues in IDC patients. The high co-expression of MAP1LC3B and SQSTM1 was associated with better DFS survival in IDC patients. The accumulated MAP1LC3B and SQSTM1 caused by autophagy inhibition increased chemosensitivity to cancer drugs (CIS and PTX) in breast cancer cell lines. We showed that the accumulation of MAP1LC3B and SQSTM1 caused by autophagy inhibition was associated with better survival of IDC patients, providing a potentially novel therapy for IDC.

Autophagy functions are a “double-edged sword” for cancer, showing opposite functions in cancer initiation by limiting genome damage/mutation or promoting cancer-cell survival in stressed microenvironments [24]. An increasing number of studies have found that autophagy defects also result in tumor formation. Moreover, the expression of the intact autophagy genes is found to be downregulated in cancers, and the spontaneous frequency of cancer malignancies is increased due to an autophagy-related gene deficiency. For example, *Beclin1* is an essential autophagy gene, and the *Beclin1*^+/−^ genotype in vivo produces spontaneous tumors such as lung and liver tumors and mammary hyperplasia [25]. Mono-allelic deletion of beclin1 has been found in many cancers such as breast, ovarian and prostate cancers [26]. The overall survival rate of patients with BECN1-negative tumors is significantly lower compared to patients with BECN1positive tumors [27]. Furthermore, the homozygote deletion of *Atg5* increases predisposition to liver tumors within the high-penetrance mouse model [28]. Deregulating autophagy by the frameshift mutations of *Atg2B*, *Atg5*, *Atg9B* and *Atg12* is involved in cancer development [29]. Somatic point mutations of *Atg5* have been found in patients with gastric cancer, colorectal cancer and hepatocellular carcinoma [30]. These results suggest that autophagy defects might promote tumor formation.

Expressions of MAP1LC3B and SQSTM1 have been widely used to evaluate autophagy status in mammalian cells and their clinical significance in cancer patients. High MAP1LC3B expression is correlated with poor survival of patients with gastric cancer [31], OSCC [3] and other disease [8]. In contrast, cancer patients with high MAP1LC3B expression have better outcomes, such as in early-stage non-small cell lung cancer (NSCLC) [32]. Moreover, high MAP1LC3B levels are associated with reduced tumor aggressiveness in early-stage NSCLC [32]. Additionally, patients with low MAP1LC3B level have a significantly poorer prognosis than patients with high MAP1LC3B [27]. On the other hand, the accumulation of SQSTM1 is used as a marker for autophagy inhibition/defects [33]. The aberrant accumulation of SQSTM1 has been found in gastrointestinal cancer [34], prostate cancer [35], hepatocellular carcinoma [36], breast cancer [37] and lung adenocarcinoma [14], suggesting that SQSTM1 accumulation through autophagy inhibition correlates with cancer progression. In this study, we found that MAP1LC3B and SQSTM1 accumulated in IDC patients and IDC patients with the high co-expression of MAP1LC3B and SQSTM1 had better DFS, which is possibly due to these tumors becoming resistant to chemotherapy or radiation therapy [38]. Moreover, autophagy is known to be involved in chemoresistance [39]. These results implied that autophagy defects might be associated with IDC progression.

Although autophagy defects result in tumor formation, mosaic deletion of *Atg5* in mice or *Atg7* in mouse livers produces only benign hepatomas, suggesting that complete and specific autophagy deficiency promotes liver tumor initiation but restricts progression to malignant disease [28]. Similarly, loss of *Atg5* or *Atg7* in the mouse pancreas promotes benign pancreatic intraepithelial neoplasia (PanIN) formation but also prevents progression of this PanIN to malignant disease [25]. Moreover, SQSTM1 accumulation by autophagy inactivation contributes to the development of benign hepatomas in mouse models, but the underlying mechanism is not known [28]. Furthermore, autophagy defects could increase drug sensitivity for cancer therapy in different types of cancers [40]. We found that chemosensitivity increases in breast cancer cell lines treated with autophagy inhibitors. Moreover, IDC patients with autophagy defects were more likely to survive. These findings demonstrate that autophagy defects cause benign breast tumor formation but prevent poor prognosis in breast cancer patients.

Breast cancer is composed of multiple molecular subtypes such asER, PR andHER2, while triple-negative breast cancer (TNBC) lacks ER, PR and HER2 expression, leading to distinct morphologies, treatment responses and clinical outcomes [41]. Autophagy dependence in breast cancer may be subtype-dependent [26]. For example, HER2 positive tumors exhibit low levels of autophagy [42], showing low expression of Beclin1 and autophagy-related genes [43]. HER2 amplification is significantly associated with decreased BECN1expression in breast cancer tumors [44]. However, TNBC tumors exhibit a higher level of autophagy with high BECN1and MAP1LC3B expressions [45]. Thus, autophagy dependence according to molecular subtype in IDC patients requires further investigation.

The present study is subject to certain limitations. Our cell culture model indicates that autophagy inhibition sensitizes breast cancer cells to chemotherapeutic drugs, leading to better survival in IDC patients. Nevertheless, autophagic flux can be measured only in cell cultures and animal models [46], not in clinical patients [4,47]. Moreover, lysosomal enzyme defects may result in pseudo-autophagy [48]. Therefore, the possibility of lysosomal dysfunction for pseudo-autophagy in breast cancer should be further studied. Additionally, our results indicated that accumulated MAP1LC3B and SQSTM1 could be caused by defective autophagy and the autophagic defect was associated with better survival and chemosensitivity. Autophagy receptor MAP1LC3B and adaptor SQSTM1 play an important role in the recruitment of cellular components into autophagosome for bulk degradation. Therefore, the knockdown of MAP1LC3B or/and SQSTM1 might result in a defective autophagy, thus protecting cancer cells. However, MAP1LC3B is one of the autophagy receptors (such as MAP1LC3A, MAP1LC3C, GABARAP and so on) and SQSTM1 is one of the adaptors (such as NBR1, NDP52, TAX1BP1 and so on) necessary for autophagy function according to previous studies [49]. The other orthologous would compensate their function while silencing MAP1LC3B or/and SQSTM1 in cancer cells. Thus, the single knockdown of MAP1LC3B and SQSTM1 or combined knockdown of MAP1LC3B and SQSTM1 might have no significant effect on the cell function. Furthermore, the analyzed data indicated that poorly differentiated tumors have a worse prognosis in IDC patients. However, MAP1LC3B upregulation was associated with poor cell differentiation but better survival. The possible reason might be that the upregulation of lipidated MAP1LC3B alone in patients could be caused by either autophagy inhibition or induction. Thus, observing MAP3LC3B protein levels alone, it was hard to tell the association of autophagy induction or inhibition with poor differentiation. To more precisely evaluate the correlation of autophagy inhibition with prognosis, elevated protein levels of both MAP1LC3B and SQSTM1 were considered as autophagy inhibition. Our results showed that the high co-expression of MAP1LC3B and SQSTM1 as defective autophagy led to better survival in IDC patients.

## 5. Conclusions

Our results find higher protein levels of MAP1LC3B and cytoplasmic SQSTM1 in the tumor tissues of IDC patients. Moreover, IDC patients with high co-expression of MAP1LC3B and SQSTM1 had better DFS. The accumulation of MAP1LC3B and SQSTM1 resulting from autophagy defects could facilitate chemosensitivity in breast cancer cell lines. Thus, the high co-expression of MAP1LC3B and SQSTM1 might be a potential biomarker for future diagnosis and prognosis in IDC patients.

## Figures and Tables

**Table 1:** The comparison of MAP1LC3B and SQSTM1 expression between adjacent normal and tumor tissues in IDC patients.

Variables	No.	Adjacent Normal		Tumor		Z	*p*-Value *
		Mean ± SD	Median	Mean ± SD	Median		
MAP1LC3B	91	4.41 ± 2.18	5.00	5.14 ± 1.84	6.00	3.186	0.001
SQSTM1	81	0.51 ± 1.16	0.00	3.84 ± 2.24	4.00	6.979	<0.001

* *p*-values were estimated by Wilcoxon signed-rank test.

**Table 2:** Correlation of MAP1LC3B and SQSTM1expressions with clinicopathological outcomes in IDC patients.

Variables	MAP1LC3B (*n* = 274)				SQSTM1 (*n* = 256)			
	%	Mean ± SD	Median	*p*-Value	%	Mean ± SD	Median	*p*-Value
Age (yr)								
<40	16.4	4.69 ± 1.99	5.00	0.036 ^†^	17.2	4.14 ± 1.90	5.00	0.172 ^†^
40–59	56.2	5.23 ± 1.81 ^a^	6.00		55.5	4.40 ± 1.98	5.00	
≥60	27.4	4.64 ± 2.04 ^a^	5.00		27.3	3.84 ± 2.27	4.00	
Pathology stage								
I	17.9	4.59 ± 2.10	5.00	0.059 ^†^	17.6	3.93 ± 2.14	5.00	0.526 ^†^
II	47.8	5.25 ± 1.83	6.00		50.4	4.19 ± 2.11	5.00	
III	34.3	4.80 ± 1.90	5.00		32.0	4.37 ± 1.93	5.00	
pT stage								
T1	27.7	4.87 ± 1.85	5.00	0.839 ^†^	27.3	3.66 ± 2.26 ^a^	4.50	0.327 ^§^
T2	60.9	5.00 ± 1.94	6.00		62.1	4.40 ± 1.88 ^a^	5.00	
T3 + T4	11.4	5.13 ± 2.00	5.00		10.5	4.48 ± 2.28	5.00	
pN stage								
N0	41.6	4.96 ± 2.08	6.00	0.593 ^§^	42.6	4.09 ± 2.03	5.00	0.869 ^†^
N1	26.6	5.23 ± 1.63	6.00		27.7	4.28 ± 2.21	5.00	
N2	21.9	4.88 ± 1.71	5.00		20.7	4.23 ± 1.87	5.00	
N3	9.9	4.59 ± 2.36	5.00		9.0	4.43 ± 2.19	5.00	
Grading								
Well + Moderate	77.7	4.85 ± 1.97	5.00	0.045 ^‡^	77.7	4.18 ± 2.02	5.00	0.693 *
Poor	22.3	5.43 ± 1.67	6.00		22.3	4.30 ± 2.19	5.00	
Vascular invasion								
Absent	65.0	5.03 ± 1.95	6.00	0.514 *	65.2	4.22 ± 2.04	5.00	0.895 *
Present	35.0	4.88 ± 1.86	5.00		34.8	4.18 ± 2.09	5.00	
Nipple invasion								
Absent	91.2	4.97 ± 1.87	5.00	0.865 *	91.8	4.23 ± 2.02	5.00	0.508 *
Present	8.8	5.04 ± 2.40	6.00		8.2	3.86 ± 2.50	5.00	

* *p*-value were estimated by student’s *T*-test. ^‡^ *p*-value were estimated by Mann-Whitney U test. ^§^ *p*-value were estimated by Kruskal-Wallis one-way ANOVA test. † *p*-value were estimated by one-way ANOVA test. ^a^ *p* = 0.028.

**Table 3:** Correlation of MAP1LC3B and SQSTM1 expressions with survival in IDC patients.

Characteristic	No. (%)	DSS				DFS			
		CHR * (95% CI)	*p*-Value	AHR ** (95% CI)	*p*-Value	CHR * (95% CI)	*p*-Value	AHR ** (95% CI)	*p*-Value
MAP1LC3B	(*n* = 255)								
Low (0–5)	125 (49.0)	1.00		1.00		1.00		1.00	
High (6–7)	130 (51.0)	0.82 (0.57–1.18)	0.286	0.64 (0.44–0.94)	0.022	0.69 (0.47–1.01)	0.053	0.54 (0.36–0.81)	0.003
SQSTM1	(*n* = 241)								
Low (0–4)	102 (42.3)	1.00		1.00		1.00		1.00	
High (5–7)	139 (57.7)	0.82 (0.56–1.20)	0.300	0.79 (0.53–1.16)	0.227	0.85 (0.58–1.26)	0.429	0.79 (0.53–1.18)	0.244

Abbreviation: DSS, disease-specific survival; DFS, disease-free survival; CHR, crude hazard ratio; AHR, adjusted hazard ratio. CHR * were estimated by univariate Cox’s regression. AHR ** were adjusted for AJCC pathological stage (II and III vs. I), grading (III vs. I and II), incomplete or inappropriate adjuvant treatment vs. non-treatment or complete adjuvant treatment and molecular subtypes (luminal B, HER2 over-expression and basal-like vs. luminal A) by multivariate Cox’s regression.

**Table 4:** Correlation of MAP1LC3B/SQSTM1 co-expression with survival in IDC patients.

Variable	No. (%)	DSS				DFS			
		CHR (95% CI)	*p*-Value *	AHR (95% CI)	*p*-Value **	CHR (95% CI)	*p*-Value *	AHR (95% CI)	*p*-Value **
MAP1LC3B(L)/SQSTM1(L)	58 (24.9)	1.00		1.00		1.00		1.00	
Either	96 (42.2)	1.07 (0.72–1.58)	0.750	1.06 (0.72–1.57)	0.773	1.27 (0.85–1.88)	0.239	1.26 (0.84–1.87)	0.262
MAP1LC3B(H)/SQSTM1(H)	79 (33.9)	0.77 (0.50–1.18)	0.233	0.66 (0.43–1.02)	0.063	0.61 (0.38–0.95)	0.030	0.51 (0.32–0.81)	0.005

Abbreviation: DSS, disease-specific survival; DFS, disease-free survival; CHR, crude hazard ratio; AHR, adjusted hazard ratio. CHR * were estimated by univariate Cox’s regression. AHR ** were adjusted for AJCC pathological stage (II and III vs. I), grading (III vs. I and II), incomplete or inappropriate adjuvant treatment vs. non-treatment or complete adjuvant treatment, and molecular subtypes (luminal B, HER2 over-expression and basal-like vs. luminal A) by multivariate Cox’s regression.

## Data Availability

Not applicable.

## Supplementary Materials

The following are available online at https://www.mdpi.com/article/10.3390/biomedicines9111514/s1, Figure S1: Effects of autophagy inhibition on sphere cell viability in breast cancer cell lines treating cancer drugs.
