# Supplementary material for: Combined Evaluation of MAP1LC3B and SQSTM1 for Biological and Clinical Significance in Ductal Carcinoma of Breast Cancer
Source: Biomedicines. 2021 Oct 21;9(11):1514. doi: 10.3390/biomedicines9111514 (PMC8615094; doi:10.3390/biomedicines9111514)
Supplement: Supplementary file 1 [file biomedicines-09-01514-s001.zip › biomedicines-1362788 supplemeantary.pdf]

Supplementary materials

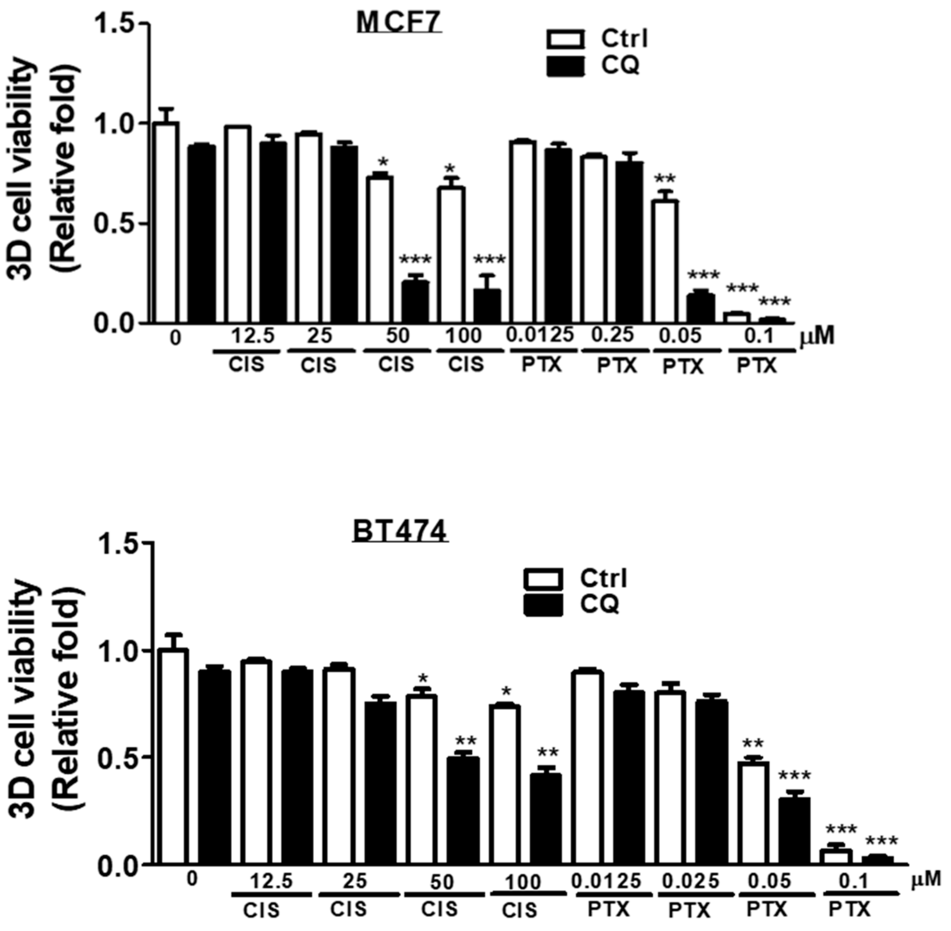

Figure S1. Effects of autophagy inhibition on sphere cell viability in breast cancer cell lines treating cancer drugs. (A) MCF7 cells and (B) BT474 cell lines pre-treated with autophagy inhibitor CQ (20 μM) for 1h were cultured in a round bottom ultra-low plate for tumorsphere formation. The tumorspheres were then treated with or without cisplatin (CIS, 0-100 μM) or paclitaxel (PTX, 0-0.1 μM) for 24 h and the sphere cell viability was measured using the CellTiterGlo 3D system. Results represent the mean of three independent experiments.
